# Editorial: Bone: Endocrine Target and Organ

**DOI:** 10.3389/fendo.2017.00354

**Published:** 2017-12-19

**Authors:** Giacomina Brunetti, Patrizia D’Amelio, Malgorzata Wasniewska, Giorgio Mori, Maria Felicia Faienza

**Affiliations:** ^1^Department of Basic and Medical Sciences, Neurosciences and Sense Organs, Section of Human Anatomy and Histology, University of Bari, Bari, Italy; ^2^Department of Medical Sciences, Gerontology Section, University of Torino, Torino, Italy; ^3^Department of Pediatrics, University of Messina, Messina, Italy; ^4^Department of Clinical and Experimental Medicine, University of Foggia, Foggia, Italy; ^5^Department of Biomedical Science and Human Oncology, Paediatric Unit, University of Bari, Bari, Italy

**Keywords:** bone and bones, hormones, PTH, immune cells, adipose tissue, glucagon-like peptide-1

**Editorial on the Research Topic**

**Bone: Endocrine Target and Organ**

Bone is essential for mobility, calcium homeostasis, and hematopoietic function. Recently, advances in bone biology have highlighted the importance of skeleton as an endocrine organ (1). In fact, hormones produced by bone cells can control mineral ion homeostasis (i.e., FGF23) and energy balance (i.e., osteocalcin). Naturally, bone is the target organ of different endocrine glands or tissues through numerous hormones and/or molecules as glucagon-like peptide-1 (GLP-1), PTH, adipokines as well as cytokines produced by immune cells. The articles in this topic highlight the effect of molecules released by gut, parathyroids, adipose tissues and immune system on bone remodeling.

In detail, GLP-1, produced and secreted by intestinal enteroendocrine L-cells, can enhance bone mineral density and improve bone quality but the specific mechanism and related molecular pathways are still not completely understood (2–5). Zhao et al. focused on the current state of research into the impact of GLP-1 on bone metabolism reporting that GLP-1 increases the number of osteoblasts and promotes the expression of genes related to bone formation (6). Zhao et al. also described that GLP-1 is also associated with increased serum levels of bone formation markers, including alkaline phosphatase, osteocalcin, and N-terminal propeptide of type I procollagen (P1NP). In general, GLP-1 may inhibit osteoclastic bone resorption. However, the specific molecular mechanisms responsible for the effects of GLP-1 have still not been fully elucidated.

Our special issue also included a review of the management of primary hyperparathyroidism (PHPT), a disease characterized by chronic overproduction of PTH. This hormone is a key regulator of bone remodeling and its overproduction is a common cause of bone loss, causes osteoporosis and is an independent risk factor for fractures. This clinical condition is of wide interest for both pathophysiological mechanisms leading to bone loss and clinical aspects of the disease; as regards pathophysiology of bone loss, an increase of the inflammatory cytokine IL-17 during PHPT in humans and in mice has been recently described (7). A clinical debate point is on the efficacy and indication for treatment of PHPT in asymptomatic disease. In this special issue, Leere et al. and Leere et al. by a systematic review provide an overview of the existing literature on contemporary pharmaceutical options available for the medical management of PHPT. The reviews by Leere et al. and Leere et al. illustrate the strengths and drawbacks of the pharmaceutical agents that are available at present and highlight a significant decrease of effect on plasma calcium both for bisphosphonates and, with different timing, for cinacalcet. The latter did not seem to have major effects on bone density and turnover, whereas long-term treatment with bisphosphonates seemed to increase bone density.

Recently, numerous researchers deepened bone and adipose tissue interactions because numerous adipokines regulated bone remodeling in physiological and pathological conditions. The research article provided in this special issue by Lecka-Czernik et al. further highlighted this issue. Marrow adipose tissue (MAT) is distinctive with respect to origin, function and metabolism. MAT has high heterogeneity which is linked to skeletal location and bone metabolism. MAT has features of both white (WAT)- and brown (BAT)-like or beige adipose tissue. Lecka-Czernik et al. reported that MAT near the trabecular bone of proximal tibia (pMAT) expressed high levels of beige fat markers, with respect to MAT located in distal tibia (dMAT). The same authors also found that in males higher trabecular bone mass is related to lower pMAT volume and higher expression of beige markers in the same location, with respect to females. Ovariectomy resulted in reduced cortical and trabecular bone mass and augmented pMAT and dMAT volume. Otherwise in males, orchiectomy determined cortical and trabecular bone loss and the trend to enlarged volume of both pMAT and dMAT.

Bone-immune system cross-talk also attracts numerous scientists (8, 9) and Leere et al. Persistent systemic inflammation can lead to heterotopic ossification, a disease lacking of successful treatment strategies. In this preliminary study, in the saliva, Sung Hsieh et al. characterized the diagnostic potential of MCP-1 and VEGF cytokines that may serve as biomarkers for an early stage diagnosis of heterotopic ossification.

In conclusion, all the articles provided a comprehensive overview of the mechanisms regulating bone remodeling. It is fundamental in order to highlight the pathophysiology of bone disease and thus identify new therapeutic targets.

## Author Contributions

All authors equally contributed to the development of this editorial.

## Conflict of Interest Statement

The authors declare that the research was conducted in the absence of any commercial or financial relationships that could be construed as a potential conflict of interest.
